# 5-Cyclo­pentyl-2-(4-fluoro­phen­yl)-3-methyl­sulfinyl-1-benzofuran

**DOI:** 10.1107/S1600536811036087

**Published:** 2011-09-14

**Authors:** Pil Ja Seo, Hong Dae Choi, Byeng Wha Son, Uk Lee

**Affiliations:** a Department of Chemistry, Dongeui University, San 24 Kaya-dong Busanjin-gu, Busan 614-714, Republic of Korea; b Department of Chemistry, Pukyong National University, 599-1 Daeyeon 3-dong, Nam-gu, Busan 608-737, Republic of Korea

## Abstract

In the title compound, C_20_H_19_FO_2_S, the cyclo­pentyl ring adopts an envelope conformation. The 4-fluoro­phenyl ring makes a dihedral angle of 27.10 (7)° with the mean plane of the benzofuran fragment. In the crystal, mol­ecules are linked by weak inter­molecular C—H⋯O hydrogen bonds and C—H⋯π inter­actions. In the cyclo­pentyl ring, one C atom is disordered over two orientations with site-occupancy factors of 0.617 (7) and 0.383 (7).

## Related literature

For the pharmacological activity of benzofuran compounds, see: Aslam *et al.* (2009 ▶); Galal *et al.* (2009 ▶); Khan *et al.* (2005 ▶). For natural products with benzofuran rings, see: Akgul & Anil (2003 ▶); Soekamto *et al.* (2003 ▶). For structural studies of related 2-(4-fluoro­phen­yl)-3-methyl­sulfinyl-1-benzofuran derivatives, see: Choi *et al.* (2010 ▶, 2011 ▶).
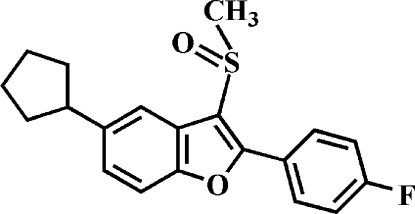


## Experimental

### 

#### Crystal data

C_20_H_19_FO_2_S
*M* *_r_* = 342.41
Orthorhombic, 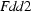

*a* = 20.0254 (13) Å
*b* = 33.197 (2) Å
*c* = 10.0233 (7) Å
*V* = 6663.3 (8) Å^3^
*Z* = 16
Mo *K*α radiation
μ = 0.21 mm^−1^
*T* = 173 K
0.35 × 0.26 × 0.19 mm

#### Data collection

Bruker SMART APEXII CCD diffractometer
Absorption correction: multi-scan (*SADABS*; Bruker, 2009 ▶) *T*_min_ = 0.930, *T*_max_ = 0.961
16962 measured reflections
4136 independent reflections
3915 reflections with *I* > 2σ(*I*)
*R*_int_ = 0.034

#### Refinement

*R*[*F* ^2^ > 2σ(*F* ^2^)] = 0.041
*wR*(*F* ^2^) = 0.106
*S* = 1.06
4136 reflections
223 parameters
96 restraints
H-atom parameters constrained
Δρ_max_ = 0.48 e Å^−3^
Δρ_min_ = −0.37 e Å^−3^
Absolute structure: Flack (1983 ▶), 1949 Friedel pairs
Flack parameter: 0.15 (7)

### 

Data collection: *APEX2* (Bruker, 2009 ▶); cell refinement: *SAINT* (Bruker, 2009 ▶); data reduction: *SAINT*; program(s) used to solve structure: *SHELXS97* (Sheldrick, 2008 ▶); program(s) used to refine structure: *SHELXL97* (Sheldrick, 2008 ▶); molecular graphics: *ORTEP-3* (Farrugia, 1997 ▶) and *DIAMOND* (Brandenburg, 1998 ▶); software used to prepare material for publication: *SHELXL97*.

## Supplementary Material

Crystal structure: contains datablock(s) I. DOI: 10.1107/S1600536811036087/gk2398sup1.cif

Structure factors: contains datablock(s) I. DOI: 10.1107/S1600536811036087/gk2398Isup2.hkl

Supplementary material file. DOI: 10.1107/S1600536811036087/gk2398Isup3.cml

Additional supplementary materials:  crystallographic information; 3D view; checkCIF report

## Figures and Tables

**Table 1:** Hydrogen-bond geometry (Å, °) *Cg* is the centroid of the C1/C2/C7/O/C8 furan ring.

*D*—H⋯*A*	*D*—H	H⋯*A*	*D*⋯*A*	*D*—H⋯*A*
C20—H20*B*⋯O2^i^	0.98	2.29	3.262 (3)	169
C16—H16⋯*Cg*^i^	0.95	2.53	3.365 (3)	146

Symmetry code: (i) 
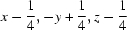
.

**Fig. 1.**
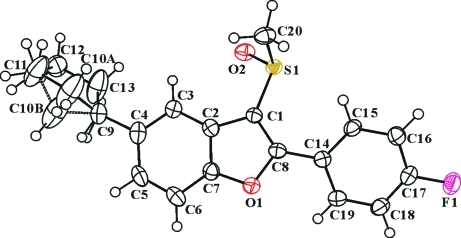
The molecular structure of the title compound with the atom numbering scheme. Displacement ellipsoids are drawn at the 50% probability level. H atoms are presented as small spheres of arbitrary radius. The C10 atom of the cyclopentyl ring is disordered over two positions with site occupancy factors, from refinement of 0.617 (7 ) (part A) and 0.383 (7) (part B).

**Fig. 2.**
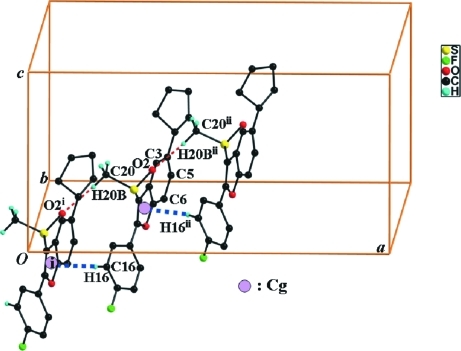
A view of the C—H···O and C—H···π interactions (dotted lines) in the crystal structure of the title compound. [Symmetry codes: (i) x -1/4 , - y + 1/4 , z - 1/4; (ii) x - 1/4, - y + 1/4, z - 1/4..]

## supplementary crystallographic information

### Comment

Many compounds containing a benzofuran ring system have attracted much interest owing to their valuable pharmacological properties such as antibacterial and antifungal, antitumor and antiviral, and antimicrobial activities (Aslam *et al.*, 2009, Galal *et al.*, 2009, Khan *et al.*, 2005). These benzofuran derivatives occur in a wide range of natural products (Akgul & Anil, 2003; Soekamto *et al.*, 2003). As a part of our ongoing study of the substituent effect on the solid state structures of 2-(4-fluorophenyl)-3-methylsulfinyl-1-benzofuran analogues (Choi *et al.*, 2010, 2011), we report herein the crystal structure of the title compound.

The title compound crystallizes ins the non-centrosymmetric space group *F*dd2. The crystal studied was an inversion twin with a 0.85 (7) : 0.15 (7) domain ratio.

In the title molecule (Fig. 1), the benzofuran unit is essentially planar, with a mean deviation of 0.024 (2) Å from the least-squares plane defined by the nine constituent atoms. The cyclopentyl ring is in the envelope form. In the cyclopentyl ring, the C10 atom is disordered over two positions with site-occupancy factors, from refinement of 0.617 (7) (part A) and 0.383 (7) (part B). The dihedral angle formed by the 4-fluorophenyl ring and the mean plane of the benzofuran fragment is 27.10 (7)°. The crystal packing (Fig. 2) is stabilized by weak intermolecular C—H···O hydrogen bonds between a methyl H atom and the O atom of the sulfinyl group (Table 1; C20—H20B···O2^i^). The crystal packing (Fig. 2) is further stabilized by intermolecular C—H···π interactions between a 4-fluorophenyl H atom and the furan ring (Table 1; C16—H16···Cg^i^, Cg is the centroid of the C1/C2/C7/O1/C8 furan ring).

### Experimental

77% 3-chloroperoxybenzoic acid (224 mg, 1.0 mmol) was added in small portions to a stirred solution of 5-cyclopentyl-2-(4-fluorophenyl)-3-methylsulfanyl-1-benzofuran (293 mg, 0.9 mmol) in dichloromethane (30 mL) at 273 K. After being stirred at room temperature for 4h, the mixture was washed with saturated sodium bicarbonate solution and the organic layer was separated, dried over magnesium sulfate, filtered and concentrated at reduced pressure. The residue was purified by column chromatography (hexane–ethyl acetate, 1:2 v/v) to afford the title compound as a colorless solid [yield 72%, m.p. 419-420 K; Rf = 0.67 (hexane–ethyl acetate, 2:1 v/v)]. Single crystals suitable for X-ray diffraction were prepared by slow evaporation of a solution of the title compound in ethyl acetate at room temperature.

### Refinement

The reported Flack parameter was obtained by TWIN/BASF procedure in SHELXL (Sheldrick, 2008). All H atoms were positioned geometrically and refined using a riding model, with C—H = 0.95 Å for aryl, 1.00 Å for methine, and 0.99 Å for methylene and methyl H atoms, respectively. *U*_iso_(H) = 1.2*U*_eq_(C) for aryl, methine, methylene, and 1.5*U*_eq_(C) for methyl H atoms. One of the C atoms of the cyclopentyl ring is disordered over two positions with site occupancy factors, from refinement of 0.617 (7) (part A) and 0.383 (7) (part B). The distances of equivalent C9—C10A and C9—C10B, and C11—C10A and C11—C10B pairs were restrained to 1.525 (3) Å, 0.001 Å and 0.001 Å using command DFIX, SADI and DELU respectively, and displacement ellipsoids of C10 set were restrained to 0.01 using command ISOR.

### Crystal data

**Table:**

C_20_H_19_FO_2_S	*F*(000) = 2880
*M**_r_* = 342.41	*D*_x_ = 1.365 Mg m^−^^3^
Orthorhombic, *F**d**d*2	Mo *K*α radiation, λ = 0.71073 Å
Hall symbol: F 2 -2d	Cell parameters from 6619 reflections
*a* = 20.0254 (13) Å	θ = 2.4–27.7°
*b* = 33.197 (2) Å	µ = 0.21 mm^−^^1^
*c* = 10.0233 (7) Å	*T* = 173 K
*V* = 6663.3 (8) Å^3^	Block, colourless
*Z* = 16	0.35 × 0.26 × 0.19 mm

### Data collection

**Table:**

Bruker SMART APEXII CCD diffractometer	4136 independent reflections
Radiation source: rotating anode	3915 reflections with *I* > 2σ(*I*)
graphite multilayer	*R*_int_ = 0.034
Detector resolution: 10.0 pixels mm^-1^	θ_max_ = 28.3°, θ_min_ = 2.4°
φ and ω scans	*h* = −26→23
Absorption correction: multi-scan (*SADABS*; Bruker, 2009)	*k* = −44→42
*T*_min_ = 0.930, *T*_max_ = 0.961	*l* = −13→13
16962 measured reflections	

### Refinement

**Table:**

Refinement on *F*^2^	Secondary atom site location: difference Fourier map
Least-squares matrix: full	Hydrogen site location: difference Fourier map
*R*[*F*^2^ > 2σ(*F*^2^)] = 0.041	H-atom parameters constrained
*wR*(*F*^2^) = 0.106	*w* = 1/[σ^2^(*F*_o_^2^) + (0.0576*P*)^2^ + 8.9505*P*] where *P* = (*F*_o_^2^ + 2*F*_c_^2^)/3
*S* = 1.06	(Δ/σ)_max_ < 0.001
4136 reflections	Δρ_max_ = 0.48 e Å^−^^3^
223 parameters	Δρ_min_ = −0.37 e Å^−^^3^
96 restraints	Absolute structure: Flack (1983), 1949 Friedel pairs
Primary atom site location: structure-invariant direct methods	Flack parameter: 0.15 (7)

### Special details

**Table:**

Geometry. All esds (except the esd in the dihedral angle between two l.s. planes) are estimated using the full covariance matrix. The cell esds are taken into account individually in the estimation of esds in distances, angles and torsion angles; correlations between esds in cell parameters are only used when they are defined by crystal symmetry. An approximate (isotropic) treatment of cell esds is used for estimating esds involving l.s. planes.
Refinement. Refinement of F^2^ against ALL reflections. The weighted R-factor wR and goodness of fit S are based on F^2^, conventional R-factors R are based on F, with F set to zero for negative F^2^. The threshold expression of F^2^ > 2sigma(F^2^) is used only for calculating R-factors(gt) etc. and is not relevant to the choice of reflections for refinement. R-factors based on F^2^ are statistically about twice as large as those based on F, and R- factors based on ALL data will be even larger.

### Fractional atomic coordinates and isotropic or equivalent isotropic displacement parameters (Å^2^)

**Table:**

	*x*	*y*	*z*	*U*_iso_*/*U*_eq_	Occ. (<1)
S1	0.28603 (2)	0.120706 (14)	0.30091 (12)	0.02721 (11)	
F1	0.22523 (7)	0.07977 (4)	−0.34573 (18)	0.0441 (3)	
O1	0.31307 (7)	0.21374 (4)	0.06022 (18)	0.0312 (3)	
O2	0.33631 (8)	0.11882 (5)	0.4113 (2)	0.0370 (4)	
C1	0.29639 (10)	0.16799 (6)	0.2227 (2)	0.0262 (4)	
C2	0.32275 (10)	0.20417 (6)	0.2840 (2)	0.0290 (4)	
C3	0.34184 (12)	0.21599 (7)	0.4113 (2)	0.0357 (5)	
H3	0.3357	0.1984	0.4851	0.043*	
C4	0.37027 (13)	0.25424 (8)	0.4294 (3)	0.0404 (5)	
C5	0.37902 (12)	0.27952 (7)	0.3188 (3)	0.0396 (5)	
H5	0.3988	0.3052	0.3320	0.048*	
C6	0.36010 (12)	0.26866 (7)	0.1916 (3)	0.0374 (5)	
H6	0.3658	0.2862	0.1175	0.045*	
C7	0.33230 (11)	0.23083 (6)	0.1784 (2)	0.0305 (4)	
C8	0.29208 (10)	0.17501 (6)	0.0893 (2)	0.0274 (4)	
C9	0.39134 (15)	0.26922 (8)	0.5643 (3)	0.0533 (7)	
H9A	0.4160	0.2951	0.5521	0.064*	0.617 (7)
H9B	0.4273	0.2883	0.5361	0.064*	0.383 (7)
C10A	0.33241 (16)	0.27685 (18)	0.6576 (2)	0.0594 (11)	0.617 (7)
H10A	0.3050	0.2997	0.6255	0.071*	0.617 (7)
H10B	0.3038	0.2526	0.6644	0.071*	0.617 (7)
C10B	0.3491 (5)	0.2980 (3)	0.64769 (16)	0.0594 (11)	0.383 (7)
H10C	0.3011	0.2945	0.6278	0.071*	0.383 (7)
H10D	0.3617	0.3263	0.6298	0.071*	0.383 (7)
C11	0.3643 (2)	0.28668 (11)	0.79210 (19)	0.0710 (10)	
H11A	0.3745	0.3158	0.7992	0.085*	0.617 (7)
H11B	0.3346	0.2789	0.8667	0.085*	0.617 (7)
H11C	0.3704	0.3113	0.8466	0.085*	0.383 (7)
H11D	0.3269	0.2709	0.8301	0.085*	0.383 (7)
C12	0.42736 (18)	0.26191 (9)	0.7924 (3)	0.0585 (7)	
H12A	0.4253	0.2407	0.8618	0.070*	
H12B	0.4669	0.2791	0.8092	0.070*	
C13	0.4306 (2)	0.24326 (13)	0.6533 (4)	0.0819 (13)	
H13A	0.4775	0.2418	0.6223	0.098*	
H13B	0.4118	0.2157	0.6546	0.098*	
C14	0.27300 (10)	0.15066 (6)	−0.0241 (2)	0.0271 (4)	
C15	0.22789 (11)	0.11913 (7)	−0.0111 (2)	0.0312 (4)	
H15	0.2076	0.1141	0.0730	0.037*	
C16	0.21211 (11)	0.09501 (7)	−0.1192 (2)	0.0338 (4)	
H16	0.1819	0.0731	−0.1099	0.041*	
C17	0.24099 (11)	0.10340 (7)	−0.2402 (2)	0.0318 (4)	
C18	0.28529 (11)	0.13457 (7)	−0.2591 (2)	0.0349 (5)	
H18	0.3042	0.1397	−0.3443	0.042*	
C19	0.30136 (11)	0.15821 (7)	−0.1500 (2)	0.0326 (4)	
H19	0.3320	0.1798	−0.1602	0.039*	
C20	0.20733 (10)	0.13041 (8)	0.3791 (3)	0.0356 (5)	
H20A	0.1952	0.1075	0.4359	0.053*	
H20B	0.1730	0.1342	0.3105	0.053*	
H20C	0.2107	0.1548	0.4337	0.053*	

### Atomic displacement parameters (Å^2^)

**Table:**

	*U*^11^	*U*^22^	*U*^33^	*U*^12^	*U*^13^	*U*^23^
S1	0.0302 (2)	0.0261 (2)	0.0254 (2)	0.00067 (19)	−0.00003 (18)	0.00238 (18)
F1	0.0492 (8)	0.0511 (8)	0.0321 (7)	−0.0062 (6)	−0.0020 (6)	−0.0098 (6)
O1	0.0371 (8)	0.0276 (7)	0.0290 (7)	−0.0008 (6)	0.0008 (6)	0.0027 (6)
O2	0.0312 (8)	0.0433 (9)	0.0365 (8)	0.0021 (7)	−0.0056 (6)	0.0111 (7)
C1	0.0253 (9)	0.0257 (9)	0.0277 (9)	0.0004 (7)	0.0014 (8)	0.0018 (7)
C2	0.0262 (9)	0.0265 (9)	0.0344 (10)	0.0029 (7)	0.0022 (8)	−0.0022 (7)
C3	0.0411 (12)	0.0342 (11)	0.0319 (10)	−0.0022 (9)	0.0019 (9)	−0.0036 (9)
C4	0.0419 (13)	0.0368 (11)	0.0425 (12)	−0.0004 (9)	0.0001 (10)	−0.0133 (9)
C5	0.0400 (12)	0.0279 (10)	0.0510 (14)	−0.0023 (9)	0.0008 (10)	−0.0085 (10)
C6	0.0383 (12)	0.0247 (9)	0.0492 (13)	0.0006 (8)	0.0032 (10)	0.0004 (8)
C7	0.0301 (10)	0.0277 (9)	0.0336 (10)	0.0036 (8)	0.0002 (7)	−0.0022 (8)
C8	0.0239 (9)	0.0288 (9)	0.0294 (10)	0.0018 (7)	0.0029 (7)	0.0033 (7)
C9	0.0850 (19)	0.0369 (11)	0.0381 (11)	−0.0150 (12)	0.0014 (12)	−0.0100 (10)
C10A	0.086 (2)	0.053 (3)	0.0391 (13)	0.0215 (19)	−0.0073 (13)	0.0017 (14)
C10B	0.086 (2)	0.053 (3)	0.0391 (13)	0.0215 (19)	−0.0073 (13)	0.0017 (14)
C11	0.108 (3)	0.071 (2)	0.0337 (10)	0.0304 (18)	−0.0064 (14)	−0.0035 (12)
C12	0.087 (2)	0.0503 (15)	0.0382 (13)	0.0047 (14)	−0.0166 (15)	0.0034 (12)
C13	0.086 (3)	0.096 (3)	0.064 (2)	0.046 (2)	−0.032 (2)	−0.032 (2)
C14	0.0247 (9)	0.0303 (9)	0.0261 (9)	0.0018 (7)	−0.0001 (7)	0.0017 (7)
C15	0.0275 (10)	0.0405 (11)	0.0256 (10)	−0.0026 (8)	0.0026 (8)	−0.0002 (8)
C16	0.0300 (10)	0.0366 (11)	0.0348 (11)	−0.0049 (8)	−0.0005 (8)	−0.0014 (9)
C17	0.0287 (10)	0.0396 (11)	0.0271 (10)	0.0032 (8)	−0.0026 (8)	−0.0040 (8)
C18	0.0359 (11)	0.0436 (12)	0.0253 (10)	0.0000 (9)	0.0018 (8)	0.0016 (9)
C19	0.0324 (11)	0.0368 (10)	0.0284 (9)	−0.0028 (8)	0.0031 (8)	0.0054 (8)
C20	0.0258 (10)	0.0486 (12)	0.0324 (11)	−0.0019 (9)	0.0010 (8)	0.0075 (9)

### Geometric parameters (Å, °)

**Table:**

S1—O2	1.4973 (16)	C10B—C11	1.5258 (14)
S1—C1	1.767 (2)	C10B—H10C	0.9900
S1—C20	1.789 (2)	C10B—H10D	0.9900
F1—C17	1.354 (2)	C11—C12	1.507 (5)
O1—C7	1.369 (3)	C11—H11A	0.9900
O1—C8	1.384 (2)	C11—H11B	0.9900
C1—C8	1.360 (3)	C11—H11C	0.9900
C1—C2	1.449 (3)	C11—H11D	0.9900
C2—C3	1.388 (3)	C12—C13	1.527 (4)
C2—C7	1.393 (3)	C12—H12A	0.9900
C3—C4	1.403 (3)	C12—H12B	0.9900
C3—H3	0.9500	C13—H13A	0.9900
C4—C5	1.402 (4)	C13—H13B	0.9900
C4—C9	1.501 (4)	C14—C15	1.389 (3)
C5—C6	1.378 (4)	C14—C19	1.406 (3)
C5—H5	0.9500	C15—C16	1.384 (3)
C6—C7	1.380 (3)	C15—H15	0.9500
C6—H6	0.9500	C16—C17	1.373 (3)
C8—C14	1.447 (3)	C16—H16	0.9500
C9—C13	1.469 (5)	C17—C18	1.376 (3)
C9—C10B	1.5249 (14)	C18—C19	1.384 (3)
C9—C10A	1.5272 (14)	C18—H18	0.9500
C9—H9A	1.0000	C19—H19	0.9500
C9—H9B	1.0000	C20—H20A	0.9800
C10A—C11	1.5266 (14)	C20—H20B	0.9800
C10A—H10A	0.9900	C20—H20C	0.9800
C10A—H10B	0.9900		
			
O2—S1—C1	106.63 (9)	C12—C11—C10A	103.6 (3)
O2—S1—C20	106.03 (10)	C12—C11—H11A	111.0
C1—S1—C20	97.93 (10)	C10B—C11—H11A	82.5
C7—O1—C8	106.75 (15)	C10A—C11—H11A	111.0
C8—C1—C2	107.34 (18)	C12—C11—H11B	111.0
C8—C1—S1	125.54 (16)	C10B—C11—H11B	131.4
C2—C1—S1	126.22 (16)	C10A—C11—H11B	111.0
C3—C2—C7	118.73 (19)	H11A—C11—H11B	109.0
C3—C2—C1	136.4 (2)	C12—C11—H11C	110.2
C7—C2—C1	104.73 (18)	C10B—C11—H11C	110.2
C2—C3—C4	119.1 (2)	C10A—C11—H11C	135.7
C2—C3—H3	120.4	H11B—C11—H11C	82.8
C4—C3—H3	120.4	C12—C11—H11D	110.2
C5—C4—C3	119.3 (2)	C10B—C11—H11D	110.2
C5—C4—C9	118.6 (2)	C10A—C11—H11D	84.9
C3—C4—C9	122.0 (2)	H11A—C11—H11D	130.1
C6—C5—C4	122.7 (2)	H11C—C11—H11D	108.5
C6—C5—H5	118.6	C11—C12—C13	104.8 (2)
C4—C5—H5	118.6	C11—C12—H12A	110.8
C5—C6—C7	116.0 (2)	C13—C12—H12A	110.8
C5—C6—H6	122.0	C11—C12—H12B	110.8
C7—C6—H6	122.0	C13—C12—H12B	110.8
O1—C7—C6	125.0 (2)	H12A—C12—H12B	108.9
O1—C7—C2	110.83 (17)	C9—C13—C12	107.1 (3)
C6—C7—C2	124.1 (2)	C9—C13—H13A	110.3
C1—C8—O1	110.33 (18)	C12—C13—H13A	110.3
C1—C8—C14	133.92 (19)	C9—C13—H13B	110.3
O1—C8—C14	115.71 (17)	C12—C13—H13B	110.3
C13—C9—C4	120.2 (2)	H13A—C13—H13B	108.6
C13—C9—C10B	109.4 (2)	C15—C14—C19	118.79 (19)
C4—C9—C10B	123.1 (3)	C15—C14—C8	121.27 (18)
C13—C9—C10A	98.0 (3)	C19—C14—C8	119.93 (18)
C4—C9—C10A	112.9 (2)	C16—C15—C14	120.7 (2)
C13—C9—H9A	108.3	C16—C15—H15	119.6
C4—C9—H9A	108.3	C14—C15—H15	119.6
C10B—C9—H9A	78.6	C17—C16—C15	118.6 (2)
C10A—C9—H9A	108.3	C17—C16—H16	120.7
C13—C9—H9B	99.1	C15—C16—H16	120.7
C4—C9—H9B	99.1	F1—C17—C16	118.3 (2)
C10B—C9—H9B	99.1	F1—C17—C18	118.61 (18)
C10A—C9—H9B	128.7	C16—C17—C18	123.1 (2)
C11—C10A—C9	104.7 (2)	C17—C18—C19	117.9 (2)
C11—C10A—H10A	110.8	C17—C18—H18	121.1
C9—C10A—H10A	110.8	C19—C18—H18	121.1
C11—C10A—H10B	110.8	C18—C19—C14	120.9 (2)
C9—C10A—H10B	110.8	C18—C19—H19	119.5
H10A—C10A—H10B	108.9	C14—C19—H19	119.5
C9—C10B—C11	104.8 (2)	S1—C20—H20A	109.5
C9—C10B—H10C	110.8	S1—C20—H20B	109.5
C11—C10B—H10C	110.8	H20A—C20—H20B	109.5
C9—C10B—H10D	110.8	S1—C20—H20C	109.5
C11—C10B—H10D	110.8	H20A—C20—H20C	109.5
H10C—C10B—H10D	108.9	H20B—C20—H20C	109.5
C12—C11—C10B	107.6 (3)		
			
O2—S1—C1—C8	141.47 (18)	C5—C4—C9—C10A	111.4 (3)
C20—S1—C1—C8	−109.11 (19)	C3—C4—C9—C10A	−68.1 (4)
O2—S1—C1—C2	−26.3 (2)	C13—C9—C10A—C11	45.6 (4)
C20—S1—C1—C2	83.13 (19)	C4—C9—C10A—C11	173.3 (3)
C8—C1—C2—C3	−176.1 (2)	C10B—C9—C10A—C11	−69.7 (3)
S1—C1—C2—C3	−6.5 (4)	C13—C9—C10B—C11	−1.6 (8)
C8—C1—C2—C7	0.1 (2)	C4—C9—C10B—C11	148.3 (4)
S1—C1—C2—C7	169.66 (15)	C10A—C9—C10B—C11	69.9 (3)
C7—C2—C3—C4	−0.2 (3)	C9—C10B—C11—C12	16.7 (8)
C1—C2—C3—C4	175.5 (2)	C9—C10B—C11—C10A	−70.0 (3)
C2—C3—C4—C5	−0.3 (4)	C9—C10A—C11—C12	−32.1 (4)
C2—C3—C4—C9	179.3 (2)	C9—C10A—C11—C10B	69.6 (3)
C3—C4—C5—C6	0.8 (4)	C10B—C11—C12—C13	−25.0 (6)
C9—C4—C5—C6	−178.8 (2)	C10A—C11—C12—C13	5.7 (4)
C4—C5—C6—C7	−0.7 (4)	C4—C9—C13—C12	−164.7 (3)
C8—O1—C7—C6	176.1 (2)	C10B—C9—C13—C12	−13.9 (6)
C8—O1—C7—C2	−1.3 (2)	C10A—C9—C13—C12	−42.3 (4)
C5—C6—C7—O1	−176.9 (2)	C11—C12—C13—C9	23.9 (4)
C5—C6—C7—C2	0.2 (3)	C1—C8—C14—C15	28.6 (3)
C3—C2—C7—O1	177.75 (19)	O1—C8—C14—C15	−154.06 (19)
C1—C2—C7—O1	0.8 (2)	C1—C8—C14—C19	−150.1 (2)
C3—C2—C7—C6	0.3 (3)	O1—C8—C14—C19	27.3 (3)
C1—C2—C7—C6	−176.7 (2)	C19—C14—C15—C16	1.3 (3)
C2—C1—C8—O1	−0.9 (2)	C8—C14—C15—C16	−177.3 (2)
S1—C1—C8—O1	−170.58 (14)	C14—C15—C16—C17	−1.2 (3)
C2—C1—C8—C14	176.6 (2)	C15—C16—C17—F1	−179.84 (19)
S1—C1—C8—C14	6.9 (3)	C15—C16—C17—C18	0.4 (4)
C7—O1—C8—C1	1.4 (2)	F1—C17—C18—C19	−179.4 (2)
C7—O1—C8—C14	−176.61 (17)	C16—C17—C18—C19	0.4 (3)
C5—C4—C9—C13	−133.7 (3)	C17—C18—C19—C14	−0.3 (3)
C3—C4—C9—C13	46.8 (4)	C15—C14—C19—C18	−0.5 (3)
C5—C4—C9—C10B	79.6 (6)	C8—C14—C19—C18	178.1 (2)
C3—C4—C9—C10B	−100.0 (6)		

### Hydrogen-bond geometry (Å, °)

**Table:**

Cg is the centroid of the C1/C2/C7/O/C8 furan ring.

**Table:**

*D*—H···*A*	*D*—H	H···*A*	*D*···*A*	*D*—H···*A*
C20—H20B···O2^i^	0.98	2.29	3.262 (3)	169.
C16—H16···Cg^i^	0.95	2.53	3.365 (3)	146.

Symmetry codes: (i) *x*−1/4, −*y*+1/4, *z*−1/4.
